# Weight-Loss Induced Changes in Physical Activity and Activity Energy Expenditure in Overweight and Obese Subjects before and after Energy Restriction

**DOI:** 10.1371/journal.pone.0059641

**Published:** 2013-03-21

**Authors:** Alberto G. Bonomi, Stijn Soenen, Annelies H. C. Goris, Klaas R. Westerterp

**Affiliations:** 1 Human Biology Department, Maastricht University, Maastricht, The Netherlands; 2 Personal Health Solutions, Philips Research, Eindhoven, The Netherlands; 3 DirectLife, Philips Consumer Lifestyle, Amsterdam, The Netherlands; Pennington Biomed Research Center, United States of America

## Abstract

Activity energy expenditure (AEE) is the component of daily energy expenditure that is mainly influenced by the amount of physical activity (PA) and by the weight of the body displaced. This study aimed at analyzing the effect of weight loss on PA and AEE. The body weight and PA of 66 overweight and obese subjects were measured at baseline and after 12 weeks of 67% energy restriction. PA was measured using a tri-axial accelerometer for movement registration (Tracmor) and quantified in activity counts. Tracmor recordings were also processed using a classification algorithm to recognize 6 common activity types engaged in during the day. A doubly-labeled water validated equation based on Tracmor output was used to estimate AEE. After weight loss, body weight decreased by 13±4%, daily activity counts augmented by 9% (95% CI: +2%, +15%), and this increase was weakly associated with the decrease in body weight (R^2^ = 7%; P<0.05). After weight loss subjects were significantly (P<0.05) less sedentary (–26 min/d), and increased the time spent walking (+11 min/d) and bicycling (+4 min/d). However, AEE decreased by 0.6±0.4 MJ/d after weight loss. On average, a 2-hour/day reduction of sedentary time by increasing ambulatory and generic activities was required to restore baseline levels of AEE. In conclusion, after weight loss PA increased but the related metabolic demand did not offset the reduction in AEE due to the lower body weight. Promoting physical activity according to the extent of weight loss might increase successfulness of weight maintenance.

## Introduction

Obesity is caused by a chronic imbalance between energy intake and expenditure. It has been reported that the amount of energy expended during physical activity plays an important role in preventing weight gain [1], [2], [3] and weight re-gain after weight loss [4], [5], [6], but contradictory results have been also presented [7]. Low levels of physical activity associated with modern sedentary lifestyles have been implicated in the etiology of obesity [2], [3], [8]. Obese children and adolescents are less physically active than their normal-weight peers [9]. Similarly, obese subjects spend more time sitting and engage in less activity than age-matched lean controls [4], [10]. Despite this difference in the level of engagement in physical activity, the activity thermogenesis, also called activity energy expenditure (AEE), is similar between lean and obese individuals [4], [9], [10], [11], [12], even when appropriate adjustments are made for differences in body size [9], [12]. The reason is that AEE depends not only on physical activity, but also on the weight of the body displaced during movements. Previous studies showed that the energy cost of weight-bearing activities, such as walking [13], and of light-intensity activities [14] was proportional to body weight. This means that obese subjects expend significantly more energy than lean ones in performing the same physical task.

Understanding the relationship between obesity and physical activity is limited by the fact that physical activity is difficult to assess under free-living conditions [15]. Indeed, physical activity is a complex human behavior which is characterized by multiple factors such as intensity, duration, frequency, and type [16]. Some of the most accurate methods for quantifying physical activity in daily life are motion sensors and doubly-labeled water. Motion sensors can directly measure physical activity by recording body movement [15], [17] and can also be used in combination with classification algorithms to identify types of activities [10], [18], [19], [20]. Doubly-labeled water represents the gold-standard technique for measuring energy expenditure in daily life and, combined with information on basal metabolic rate (BMR), can be used to determine AEE in free-living conditions. However, comparing the amount of physical activity between individuals requires a correction of AEE for body size [21].

Whereas lean and obese individuals show similar levels of AEE, reduced-obese subjects have a lower AEE. This was observed in many studies analyzing the effect of physiological adaptation to energy restriction [22], [23], [24], [25], [26]. Interpreting doubly-labeled water data, Redman et al. [25] concluded that the reduced AEE following weight loss was caused by a lower cost of physical activity and by reduced physical activity. However, motion sensors have seldom been used to measure body movement before and after weight loss. They can provide a more direct measure of physical activity and could help our understanding of why reduced-obese subjects cannot adapt their behavior to reach levels of AEE similar to that of lean and obese subjects.

In this study, physical activity was measured in a population of overweight and obese subjects using a motion sensor at two different levels of body weight. An accelerometer was used to quantify the amount of physical activity as well as the individuals’ activity behavior. This accelerometer had a number of unique features. Firstly, it has been extensively validated against doubly-labeled water [15], and the measured activity counts proved to highly correlate with energy expenditure in free-living conditions [19]. In addition, a classification algorithm was developed to process the raw acceleration data for identifying daily activities such as lying, sitting or standing, actively standing, walking, bicycling, and running [18]. The aim was to investigate the effect of weight loss on physical activity and AEE, and to model which change in physical activity could offset the weight-loss induced variation in AEE.

## Methods

### Subjects

Seventy subjects were recruited to participate in this study. Inclusion criteria were age 25–70 years and BMI >27 kg/m^2^. Exclusion criteria were underlying malignity, cancer, HIV infection, psychiatric disease, more than 10% reduction in body weight during the previous 6 months, and women who were pregnant or breastfeeding. Of the 70 participants who started, 4 subjects dropped out. Two participants stopped due to personal reasons, and two were excluded from the analysis due to malfunction of the motion sensor or because of too little monitoring time of physical activity. The final study population consisted of 66 subjects, 10 males and 56 females. None of the participants reported to take any medication and 10 volunteers were diagnosed with diabetes mellitus type 2. The medical ethical committee of the University Medical Center Groningen approved the study. All participants gave written informed consent.

### Protocol

After two weeks of weight maintenance, subjects followed for 12 weeks a prescribed diet providing a 67% energy restriction from baseline energy requirements. At the end of the weight loss phase, subjects underwent another weight maintenance period of two weeks. The energy requirements for weight maintenance was calculated for each participant individually based on estimates of resting metabolism multiplied by 1.5 for total energy expenditure, assuming: 1) the ratio between total and basal energy expenditure being typically between 1.6 and 1.65 in overweight and obese subjects [12], [27]; 2) basal energy expenditure is on average 10% lower than resting metabolic rate. Resting metabolism was calculated using the Harris and Benedict equation since a recent validation study [28] showed accurate estimates (error <10%) in a consistent fraction of obese and non-obese subjects. Participants followed a standardized group-organized program guided by dietitians. The program focused on eating behavior and healthy diet. Since usual Dutch breakfasts and lunches are bread-based, breakfasts and lunches consisted of whole-meal and multi-grain bread (low glycemic index) and butter (fat) cheese, cold sliced meat, coldfish (protein and fat), marmalade, and honey (carbohydrates), and a dairy-based drink (protein and fat). Dinners consisted of boiled potatoes (carbohydrates), vegetables and meat or fish (protein and fat), with a sauce (fat), and a dairy-based dessert (protein and fat). Water and a limited amount of coffee and tea (in total of 3 cups a day without sugar) were allowed to be drunk. By adapting the relative amounts of the food-items to the necessary macronutrient compositions and the necessary percentages of energy intake the absolute amounts were obtained. Each individual received their unique menu to achieve weight loss over three months based upon 33% of their subject-specific energy requirements. Physical activity was not prescribed as part of the intervention. Participants visited the clinic every week in the first month of the weight loss program and every 2 weeks in the following 2 months, in total 9 sessions over the 3 months. Additionally, participants visited the laboratory at the beginning and end of the weight maintenance phases, preceding and following the weight loss phase. Measurements of subjects’ physical characteristics with the exception of body height were taken at each scheduled visit. Body weight (BW) was measured with subjects in underwear after an overnight fast, using a calibrated hospital scale to the nearest 0.1 kg (model BC-418, Tanita, Arlington Heights, IL). Height was measured to the nearest 0.1 cm (model 240 stadiometer, Seca, Hamburg, Germany). Baseline values were defined as the average values measured at the beginning and end of the first weight maintenance phase. Values after weight loss were defined as the average of the values measured at the beginning and end of the second weight maintenance phase. During the baseline weight-maintenance phase, body weight did not significantly change as determined using the paired t-test (mean change: –0.22 kg, CI: from –0.53 to 0.10 kg, P = 0.18). Similarly, during the weight-maintenance phase after weight loss, body weight did not significantly change (mean change: –0.08 kg, CI: from –0.32 to 0.16 kg, P = 0.50). The physical activity was monitored for 14 days during the two weight maintenance phases, thus at baseline and after weight loss.

### Physical activity and Activity Energy Expenditure

Physical activity was monitored using a tri-axial accelerometer for movement registration (Tracmor, Philips Research, Eindhoven, The Netherlands) [18], [19]. This instrument was a small 8×3.5×1 cm lightweight device (34.8 g, including battery), which was placed on the lower back of the subjects by means of an elastic belt. The Tracmor was equipped with a piezo-capacitive tri-axial accelerometer able to collect information about both the static and dynamic components of the acceleration forces acting on the sensor. This feature was helpful for distinguishing between types of physical activity and body postures by collecting specific information about the device orientation. The sampling frequency of the accelerometer was set to 20 Hz, and the device was oriented to align the x, y, and z sensing axes to the vertical, medio-lateral, and antero-posterior directions of the body respectively. The subjects were instructed to wear the Tracmor during waking hours, except when showering or during water activities. The subjects were given a diary in which to record the times when they woke up, went to sleep and took off the Tracmor belt during the day.

The Tracmor output was processed to determine total amount of body movement by measuring activity counts, as previously presented [19], [29], [30], [31], [32]. Tracmor activity counts were calculated over the monitoring period, and the sum of the counts was divided by the number of monitoring days to determine the average activity counts per day (Cnts/d) [19]. The AEE was measured using a doubly-labeled water validated equation based on Cnts/d and BW [19].

### Identification of Activity Types

**Figure 1:**
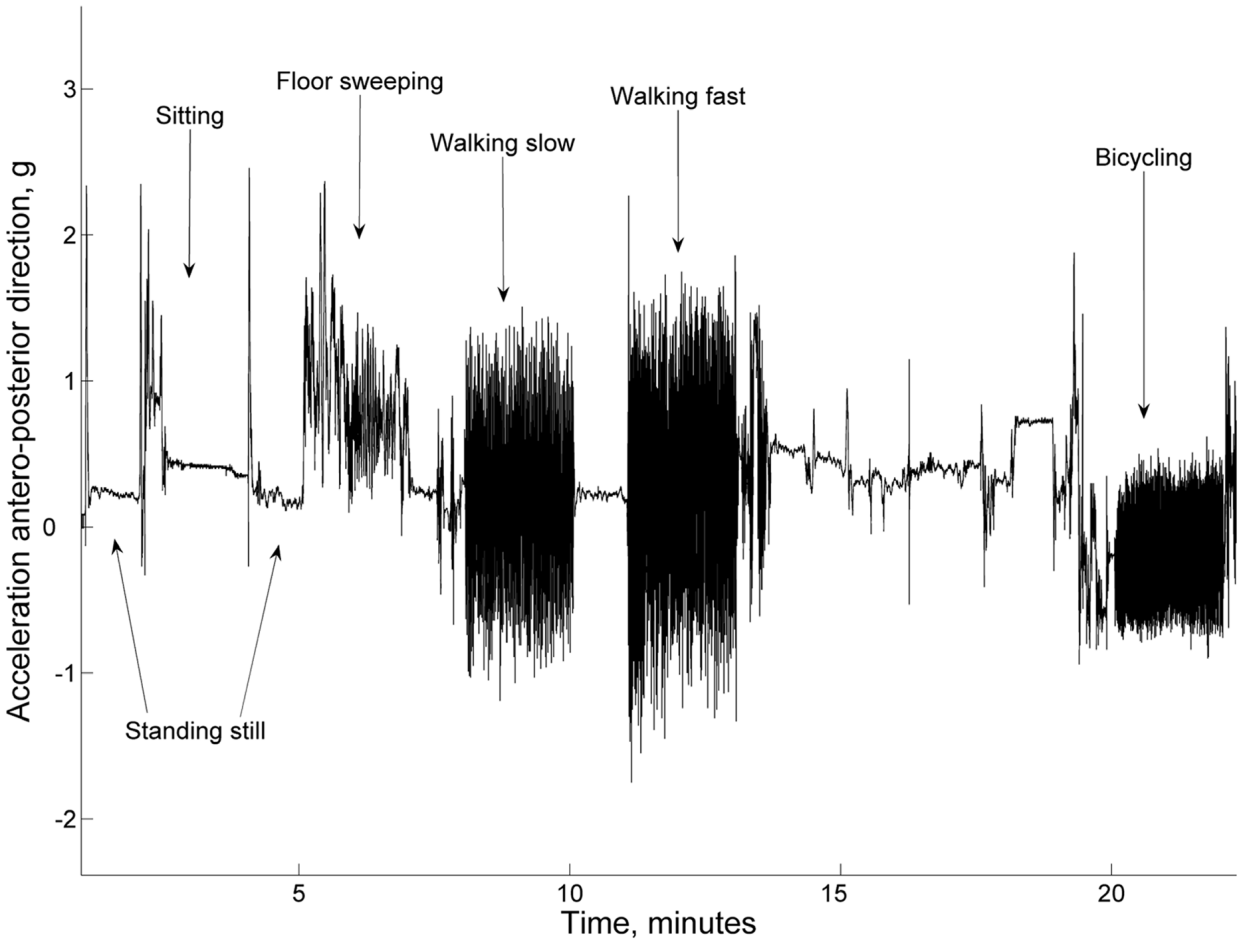
Acceleration signal measured using the tri-axial accelerometer during standardized activities and used to develop the classification tree. The signal represents the antero-posterior acceleration of the body during different activities and postures.

The types of activities subjects performed during the day were identified by analyzing the raw signal measured with the Tracmor. This process involved classifying the acceleration signal by using the knowledge contained in a machine learning algorithm. The acceleration signal was downloaded to a personal computer, segmented into intervals of 6.4 seconds, and characteristics (features) of the acceleration were measured for each axis, such as average, standard deviation, peak-to-peak distance, and dominant frequency in the power spectral density [18]. A classification tree was used to evaluate the features and classify them into one of 6 activity classes: lying, sitting or standing (sit-stand), actively standing, walking, bicycling, and running. The actively standing type was defined to represent dynamic activities not related to ambulation performed in the standing position. The classification tree was developed in a population characterized by a broad range of weight, height and age: 37 men and 43 women, (mean ± SD [min.; max.]) weight = 78±20 [51; 182] kg, height = 1.72±0.1 [1.49; 1.97] m, age = 42±16 [19; 71] years and BMI = 26.2±5.8 [19.2; 53.9] kg/m^2^. The calibration of the classification tree was based on data collected during supervised tests. These supervised tests involved activities such as lying, sitting, standing, walking, running, bicycling, washing dishes and sweeping the floor. The data collected during the dishwashing and floor-sweeping activities were used to define the actively standing category. From the acceleration signal recorded during the standardized activity trial (***Figure 1***), rules based on acceleration features were learned and used by the classification tree for identifying activity types. These rules are represented by the structure of the classification tree, and the accuracy of the classification tree was found to be on average 92% as tested in a laboratory trial for an independent study population [19]. Additionally, a previous free-living validation study showed that the assessment of walking, running, and cycling duration using the classification tree method was not significantly different from that provided by a validated multi-sensor activity monitor (IDEEA, MiniSun, Fresno, CA) augmented by diary annotations [33].

### Statistics and Data Processing

The paired t-test was used to test significant changes in the measured parameters at baseline and after weight loss. The change in variables was calculated as the difference between the after weight loss value and the baseline value. The stepwise multiple-linear regression analysis was used to identify which subjects’ physical characteristics (gender, age, BW, height, BMI) predicted the amount of body movement (Cnts/d) and the daily duration of the 6 types of activity, both at baseline and after weight loss. Additionally, stepwise multiple-linear regression was performed to select the best predictors of the change in total amount of body movement (Cnts/d) registered after weight loss. Environmental temperature and daylight hours were used as independent variables in the stepwise regression analysis to evaluate the contribution of seasonality to physical activity. The results of the regression analysis were expressed in terms of partial correlation coefficient (Partial R^2^), and regression coefficient (β) of each independent variable in the equation. The AEE doubly-labeled water prediction model, as presented in the equation below:
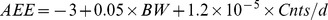
was used to determine the theoretical independent contribution of the weight loss and of the change in body movement to the change in AEE [19].

Monitoring days of physical activity were considered valid if the non-wearing time, as annotated in the diary, did not exceed 150 min/d. As a result, the average number of monitoring days was 8±3 days (range: 2–14 days) at baseline and 8±3 days (range: 2–14 days) after weight loss. The non-wearing time was removed from the dataset and not used by the classification tree for identifying activity type. For each subject, the activity behavior was defined at baseline and after weight loss by measuring the average daily duration of the sleeping, lying, sit-stand, active standing, walking, bicycling, and running activity types. The time spent sleeping was determined by the diary annotations. The lying time was determined by the duration of lying down during waking hours. The running duration was not normally distributed and therefore was log transformed for the statistical analysis. All analyses were carried out using Matlab statistical toolbox (The MathWorks, Natick MA) and SigmaStat (Systat software, San Jose CA). Data in text and tables are presented as average ± standard deviation. The statistical significance level was set to P<0.05.

## Results

**Figure 2:**
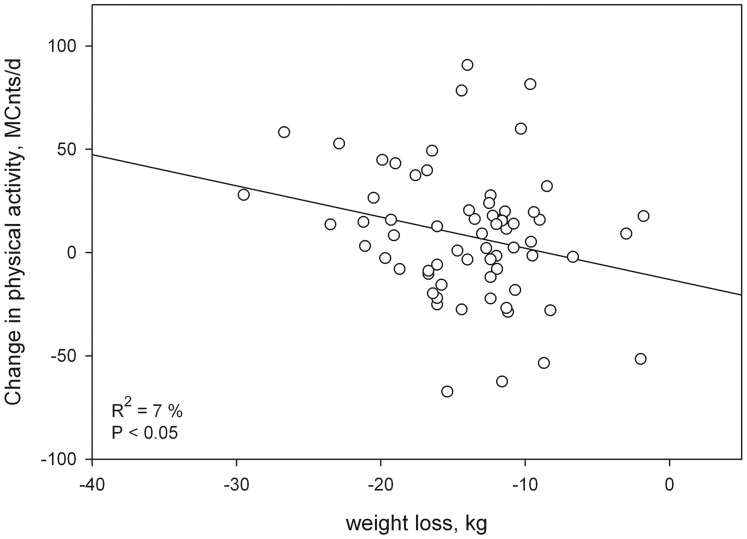
Association between the change in body movement and the change in body weight.

Subject characteristics at baseline and after weight loss are presented in ***Table 1***. BW decreased by 14±5 kg during energy restriction. This represented 13±4% of the initial BW. As would be expected from the decreased body size, the AEE estimated using the doubly-labeled water validated equation was significantly lower after weight loss. Despite the decrease in AEE, the amount of body movement was significantly higher after weight loss (***Table 1***). The measured Cnts/d increased by 9±27% (95% CI: from 2 to 15%), and this increase was weakly associated with BW change (β <0; Partial R^2^ = 7%; P<0.05) (***Figure 2***). Stepwise multiple-linear regression showed that the measured Cnts/d at baseline were negatively associated with age (β = −854; Partial R^2^ = 7%; P<0.05) and BMI (β = −957; Partial R^2^ = 7%; P<0.05). After weight loss, the measured Cnts/d were predicted by age only (β = −1333; R^2^ = 18%; P<0.05). No seasonal effect was observed in the regression equations.

**Table 1:** Subjects’ characteristics (n = 66), energy expenditure and physical activity at baseline and after weight loss.

	Baseline	After weight loss	P	95% CI
*Subjects’ characteristics*				
Sex, M/F	10/56	–		
Age, years	51±12	–		
Height, m	1.69±0.08	–		
Body weight, kg	109.5±21.1	95.6±19.6	**<0.001**	12.7, 15.1
BMI, kg/m^2^	38.3±7.1	33.4±6.3	**<0.001**	4.4, 5.2
*Energy expenditure*				
RMR, MJ/day	7.7±1.4	7.1±1.2	**<0.001**	0.5, 0.7
AEE, MJ/day	3.9±1.0	3.3±0.9	**<0.001**	0.5, 0.66
*Physical activity*				
Body movement, kCnts/day	114.1±28.9	122.2±38.1	**<0.05**	−15.8, −0.4

95% CI, confidence interval of the difference (Baseline – after weight loss); BMI, body mass index; RMR, resting metabolic rate; AEE, activity energy expenditure; Body movement, physical activity measured using the motion sensor; kCnts/day, kilo (x10^3^) counts per day.

The activity behavior was predominantly sedentary. Excluding the sleeping period, more than 51% of the time was spent lying, sitting or standing still and only 5% was spent walking. The daily duration of sitting and standing was positively associated with age (β >0; R^2^ = 11%; P<0.01), while the duration of actively standing was negatively associated with age (β <0; R^2^ = 13%; P<0.01). The engagement in walking was predicted by age (β <0; Partial R^2^ = 6%; P<0.05) and BMI (β <0; Partial R^2^ = 6%; P<0.05). Thus, once the negative contribution of age to the daily duration of walking is removed, a significant influence of BMI on the amount of time spent walking was observed. The daily duration of other activity types was not associated with any physical characteristics at baseline (***Table 2***).

**Table 2:** Relationship between the daily duration of different types of activities and subjects’ characteristics at baseline and after weight loss.

	Baseline		After weight loss	
	Equation	R^2^	Equation	R^2^
*Behaviour*				
Sleep	n.s	–	n.s.	–
Lie	n.s	–	n.s.	–
Sit-stand	250+3 age	11%	158+4 age	19%
AS	513–3 age	13%	539–3 age	18%
Walk	101–0.4 age –1.1 BMI	14%	71–0.6 age	8%
Bicycle	n.s.	–	n.s.	–
Run	n.s.	–	n.s.	–

Equation, results of the stepwise multiple linear regression between subjects’ characteristics and daily duration of each activity type; R^2^, correlation coefficient of the regression equation; Sit-stand, sitting or standing; AS, actively standing; n.s., not statistically significant.

**Figure 3:**
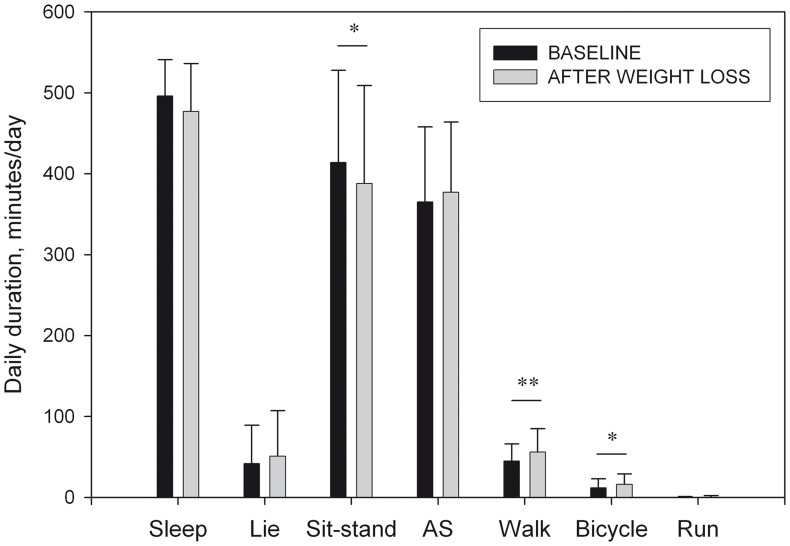
Duration of the types of activity performed at baseline and after weight loss. Sit-stand; daily duration of sitting and standing still. AS; daily duration of actively standing. (*) or (**); significant difference between baseline and after weight loss (P<0.05 or P<0.001).

The stepwise prediction models showed that, after weight loss, age was the only parameter explaining the variability in the duration of sitting and standing, actively standing, or walking; while the daily duration of sleeping, lying, bicycling and running was not associated with any physical characteristics. No seasonal effect was observed in the regression equations (***Table 2***). After weight loss the activity behavior significantly changed: subjects spent less time sitting or standing still (−26±90 min/d, P<0.05, standard error [SE] = 11.1 min/d), and more time walking (+11±21 min/d, P<0.001, SE = 2.6 min/d) and bicycling (+4±14 min/d, P<0.05, SE = 1.8 min/d) (***Figure 3***).

**Figure 4:**
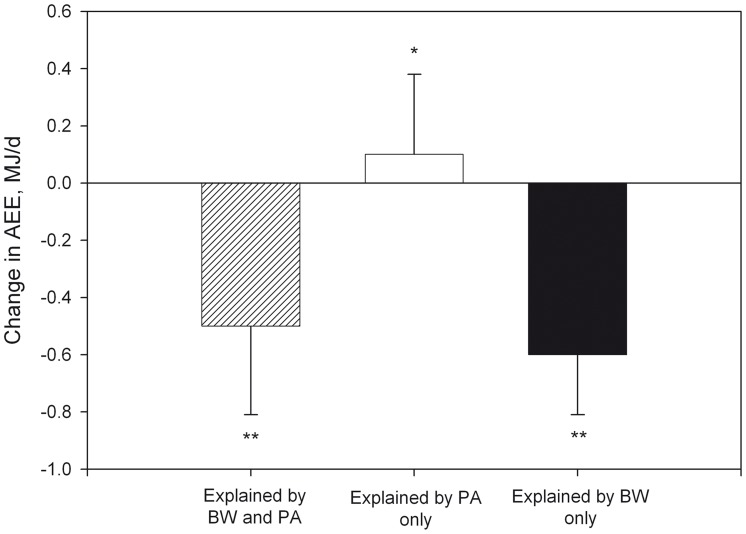
Contribution of the change in body weight (BW) and the change in physical activity (PA) to the change in activity energy expenditure (AEE). (*) or (**); significant difference from zero (P<0.05 or P<0.001).

According to the doubly-labeled water validated model, the change in AEE not accounted for by body movement, and, therefore, induced by the change in BW was –0.70±0.26 MJ/d (95% CI: from –0.76 to –0.63). The change in AEE induced by the change in body movement, i.e. not accounted for by BW, was +0.10±0.38 MJ/d (95% CI: from 0.004 to 0.19). As a result of the change in both BW and body movement, AEE significantly decreased by 0.60±0.40 MJ/d (95% CI: from –0.70 to –0.50) after weight loss (***Figure 4***). The doubly-labeled water validated equation was also used to calculate for each subject the amount of activity counts needed to obtain the baseline AEE given a body weight as measured after weight loss. Hence, to compensate for the decrease in AEE due to the change in BW, body movement should have increased by 58±21 kCnts/d (55±29% of the baseline Cnts/d, CI: from 47 to 61%).

## Discussion

Weight loss induces a reduction in AEE that hinders from achieving successful weight maintenance. Indeed, high AEE counteracts the decline of lean mass and thereby of metabolic rate which can compensate for the negative impact of poor compliance to a low-caloric diet regime [25]. This study showed a decrease in AEE following weight loss due to the low metabolic cost for carrying a smaller body weight during physical activity. In spite of this observation, a mild increase in physical activity accompanied weight loss. Reduced-obese and overweight subjects spent significantly more time walking and bicycling and less time sedentary. Thus, to preserve AEE weight-reduced subjects should improve their physical activity according to the extent of their loss in weight.

The physical activity measured at baseline and expressed as the amount of body movement was inversely associated with age and BMI. This is in line with other studies showing how physical activity decreases with age [34] and with increasing BMI [35], [36]. After weight loss, the negative effect of BMI on Cnts/d weakened. Similarly, the walking time measured at baseline was negatively associated with BMI but not after weight loss. The duration of walking periods following weight loss exceeded what predicted by age and BMI according to the model developed at baseline (56±29 min/d vs. 49±7 min/d, P<0.05). This reveals that body size can play a significant role in influencing overweight and obese subjects’ engagement in physical activity. To confirm this, the change in activity counts was associated with the amount of weight loss. This may indicate that elevated body weight could result in impaired bodily function, limiting the ability of obese subjects to perform physical tasks. A number of studies have shown how obesity and excess body weight could impose functional limitations, such as overloading the locomotive system during weight-bearing activities [37], in particular during walking [38], which could potentially limit physical activity [27]. Considering that low levels of physical activity play an important role in the development of obesity [39], these findings support the hypothesis that inactivity and the accumulation of body weight might reinforce one another in the process of developing and maintaining the overweight and obese state.

Many previous studies investigated the effect of energy restriction on physical activity in obese subjects, and the results were contradictory. Weinsier et al [40] reported that obese women tended to be more physically active after weight loss. Others reported no change in physical activity after energy restriction as measured using Doppler-radar in the confined environment of a respiration chamber [23], [24]. Accordingly, a proposed theory states that the physical activity is biologically determined and not altered by perturbations in body weight [4]. Then, when physical activity was determined using doubly-labeled water, i.e. by correcting energy expenditure for differences in body size, dieting subjects decreased their engagement in physical activity as reported by the semi-starvation Minnesota study [26] and in less severe energy restriction studies [22], [23], [24], [25]. However, interpreting doubly-labeled water data to determine physical activity is controversial. The relationship between AEE and body weight is complex as it depends on the type of activity performed [21]. The AEE resulting from weight-bearing activities, such as walking and stepping, is directly related to BW [21], but during sedentary activities and bicycling AEE is proportional to BW raised to the power of 0.3 and 0.5, respectively [21]. To further complicate the AEE vs. BW association, BW influences the amount of physical activity engaged in, and in particular the types of activity performed. This means that a unique correction factor of AEE can hardly be established to determine the amount of physical activity from doubly-labeled water data. Schoeller et al. [14] showed that BW represents a proper correction factor for AEE during light-intensity activities, because the relationship between AEE and BW has a zero intercept and a slope coefficient close to one. Other studies [21], [41] pointed out that BW raised to the power of 0.5 can be used to normalize AEE. In our study, combining the results of Prentice et al [21] with the measurements of activity behavior we observed that AEE was linearly dependent on BW raised to the power of 0.35±0.04 at baseline and raised to the power of 0.36±0.04 after weight loss. This highlights the fact that doubly-labeled water derived measurements of AEE should be carefully interpreted to determine body movement at different levels of body weight.

Obese subjects have comparable levels of AEE to lean ones [4], [10], even if they are generally less physically active [9], [10], [11], [12]. Reduced-obese subjects, because of the negative impact of weight loss on the energy cost of physical activity, have smaller AEE compared to both lean and obese subjects [22], [23], [24]. In this study, only a few individuals (n = 5) could offset the reduction in AEE due to weight loss (change in AEE was 0.29±0.15 MJ/d) by basically reducing the sedentary time by 2 hours/day and increasing the time spent actively standing, walking and bicycling by 50, 30 and 5 minutes/day respectively. This behavioral change resulted in a 59% ±27% increase in the amount of body movement (or 65±29 kCnts/d). Although motivating individuals in being more physically active remains challenge, such a modification in the activity behavior seems a realistic target for reduced-overweight and obese subjects to compensate for the lower AEE following weight loss.

The strength of this study was that free-living physical activity was measured before and after weight loss using an objective and validated method, which allowed both an assessment of the total amount of body movement and a definition of the individuals’ activity behavior. The activity classification system employed to identify activity types has been successfully validated in free-living individuals [33]. However, the lack of a reference technique to determine duration of certain activity types like free-living cycling hampers the quantification of the methodological accuracy of the classification tree. A further limitation was that energy expenditure was not actually measured using the gold standard technique of doubly-labeled water, but estimated from a prediction equation based on activity counts and subject characteristics. However, the accelerometer output has been extensively validated against doubly-labeled water and it showed to be among the most accurate activity monitors in terms of estimation error of energy expenditure [15]. Similarly, RMR was not measured but estimated from subjects’ characteristics, thus metabolic adaptations to energy restriction could not be observed. Furthermore, the concept of metabolic efficiency was not considered as a possible determinant of the change in AEE following weight loss. The reason was that currently there is no clear indication of whether weight loss could induce an increase in metabolic efficiency, as defined by the amount of energy per unit of body weight necessary for an individual to perform a certain physical task. Indeed, while a few studies [22], [24] reported changes in metabolic efficiency after weight loss, many others [9], [26], [40] disagree with the hypothesis that weight loss could result in increased metabolic efficiency. Whether less severe energy restrictions would lead to similar observations still remains to be elucidated. In addition, because of the absence of a control group it remains unclear whether the increased physical activity following diet was the result of the beneficial effect of weight loss or a cognitive modification related to the intervention.

In conclusion, exposure to physical activity is essential to improve weight maintenance. Indeed, mechanisms modulating AEE in response to fluctuations in energy intake are important to maintain body weight. However, after weight loss, due to the lower weight carried, a higher amount of body movement is required to adjust for excess in energy intake. Although a mild increase in physical activity was stimulated by weight loss in the study population, preservation of baseline AEE could not be achieved. A behavioral change equivalent to a 2-hour reduction per day of sedentary time, and an increase in ambulatory activities showed to compensate for the decline in AEE. Thus, subjects can offset the weight loss induced decrease in AEE by increasing physical activity, and this certainly contributes to the successfulness of weight maintenance after a dieting program.
